# Evaluation of technical performance of optical surface imaging system using conventional and novel stereotactic radiosurgery algorithms

**DOI:** 10.1002/acm2.13152

**Published:** 2020-12-27

**Authors:** Hironori Kojima, Akihiro Takemura, Shogo Kurokawa, Shinichi Ueda, Kimiya Noto, Haruna Yokoyama, Shigeyuki Takamatsu

**Affiliations:** ^1^ Department of Radiology Kanazawa University Hospital Kanazawa Ishikawa Japan; ^2^ Division of Health Sciences Graduate School of Medical Sciences Kanazawa University Kanazawa Ishikawa Japan; ^3^ Faculty of Health Sciences Institute of Medical, Pharmaceutical and Health Sciences Kanazawa University Kanazawa Ishikawa Japan; ^4^ Department of Radiation Technology Shizuoka General Hospital Shizuoka Shizuoka Japan; ^5^ Department of Radiation Therapy Kanazawa University Hospital Kanazawa Ishikawa Japan

**Keywords:** optical surface imaging system, positioning, registration, surface‐guided radiation therapy (SGRT), stereotactic radiosurgery (SRS)

## Abstract

The Catalyst HD (C‐RAD Positioning AB, Uppsala, Sweden) optical surface imaging (OSI) system is able to manage interfractional patient positioning, intrafractional motion monitoring, and non‐contact respiratory gating without x‐ray exposure for radiation therapy. In recent years, a novel high‐precision surface registration algorithm for stereotactic radiosurgery (SRS algorithm) has been released. This study aimed to evaluate the technical performance of the OSI system using rigid phantoms, by comparing the conventional and SRS algorithms. To determine the system’s technical performance, isocenter displacements were calculated by surface image registration via the OSI system using head, thorax, and pelvis rigid phantoms. The reproducibility of positioning was evaluated by the mean value calculated by repeating the registration 10 times, without moving each phantom. The accuracy of positioning was evaluated by the mean value of the residual error, where the 10 offset values given to each phantom were subtracted from the isocenter displacement values. The stability of motion monitoring was evaluated by measuring isocenter drift during 20 min and averaging it over 10 measurements. For the head phantom, all tests were compared with the mask types and algorithms. As a result, for all sites and both algorithms, the reproducibility, accuracy, and stability for translation and rotation were <0.1 mm and <0.1°, <1.0 mm and <1.0°, and <0.1 mm and <0.1°, respectively. In particular, the SRS algorithm had a small absolute error and standard deviation of calculated isocenter displacement, and a significantly higher reproducibility and accuracy than the conventional algorithm (*P* < 0.01). There was no difference in the stability between the algorithms (*P* = 0.0280). The SRS algorithm was found to be suitable for the treatment of rigid body sites with less deformation and small area, such as the head and face.

## 1 INTRODUCTION

In recent years, several optical surface imaging (OSI) systems have been commercialized. Surface‐guided radiation therapy^1^ is a technique which uses an OSI system to verify interfractional patient positioning, monitor intrafractional motion, and perform non‐contact respiratory gating without x‐ray exposure. The Catalyst HD (C‐RAD Positioning AB, Uppsala, Sweden) OSI system uses the optical reflectance of a projected pattern to generate a three‐dimensional surface. Through registration with the reference surface, the OSI system estimates the isocenter displacement during positioning and motion monitoring.

A novel registration algorithm of the OSI system for stereotactic radiosurgery (SRS) and stereotactic radiotherapy (SRT) of the head using an open‐type mask has been developed. The SRS algorithm uses semi‐non‐rigid registration, which is optimized to manage small surface deformations such as eye movements. Furthermore, the resolution of a surface image used for the SRS algorithm is approximately four times higher than that of a surface image used for the conventional algorithm. Therefore, the SRS algorithm can yield high‐precision registration and may detect slight isocenter displacements. Several studies have investigated Sentinel system^2^ (C‐RAD Positioning AB, Uppsala, Sweden), which is based on surface scanning with line laser light using a single scanner unit, Catalyst system^3^, ^4^ (C‐RAD Positioning AB, Uppsala, Sweden) based on surface scanning with projected light using a single scanner unit, and the conventional algorithm of the Catalyst HD system^5^ on its applicability for clinical use and technical performance. However, data on the technical performance of the SRS algorithm of the Catalyst HD system have not been published. Therefore, the SRS algorithm of Catalyst HD system should be verified by analyzing its technical performance with respect to positioning and monitoring for clinical use.

This study aims to evaluate the reproducibility and accuracy of interfractional patient positioning and the stability of intrafractional motion monitoring of the OSI system by comparing the conventional algorithm with the SRS algorithm.

## 2 MATERIALS AND METHODS

### 2.A OSI system

Catalyst HD was used as the OSI system in this study. The OSI system consists of three scanner units arranged at angles of approximately 120° to each other, to enable continuous surface detection of a patient, even if the gantry hides the patient from one of the three scanners. Each scanner unit emits visible blue light with a wavelength of 405 nm to the patient, and the projected light illuminates the patient. The reflected light is captured by the charge‐coupled device cameras. The frame rate of surface capture is 200 frames per second, and the maximal scan volume has a width, length, and height of 800, 1300, and 700 mm, respectively. The scan volume is manually adjustable through a software interface. When the scan volume is changed, the reference surface can be cropped to a smaller volume than the scan volume. In the conventional algorithm, the captured surface is registered with the cropped reference surface by using a non‐rigid iterative closest point (ICP) algorithm.^6^, ^7^ The registration identifies position errors between the captured surface and the reference surface in six degrees of freedom (DOF): three representing translations and three representing rotations.

The SRS algorithm for head SRS and SRT calculates isocenter displacements through semi‐non‐rigid ICP registration. In addition, the SRS algorithm captures a scanned image of 640 × 480 pixels from one camera unit of the OSI system as the full field of view, whereas the conventional algorithm captures an image with a resolution of 320 × 240 pixels. The surface image of the SRS algorithm consists of up to 921 600 points and that of the conventional algorithm consists of up to 230 400 points. Therefore, the spatial resolution of the SRS algorithm is approximately four times higher than that of the conventional algorithm.

### 2.B Technical performance tests of OSI system

**Fig. 1:**
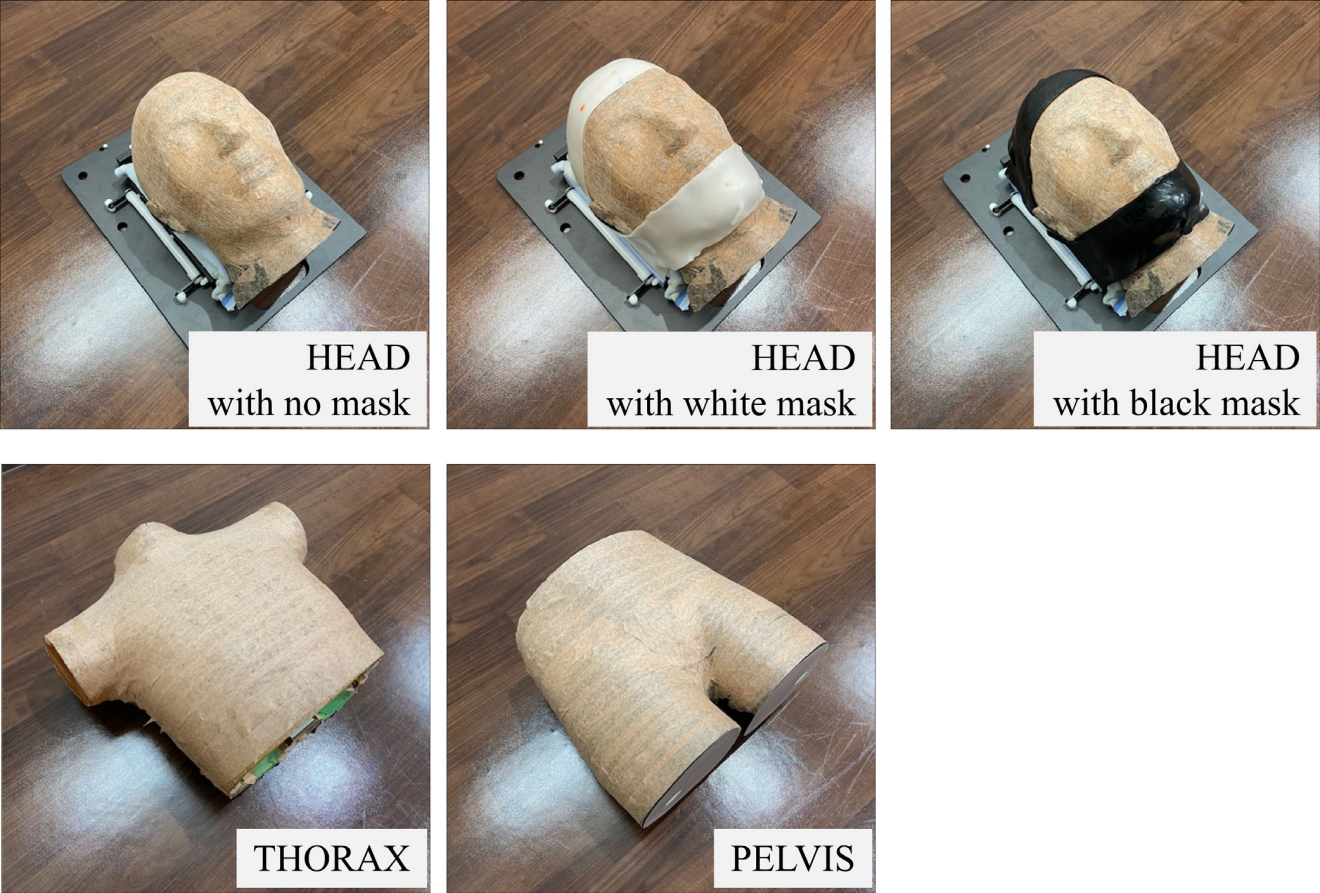
The phantoms and masks for the technical performance test.

As shown in Fig. 1, the following anthropomorphic rigid phantoms were used to test the reproducibility, accuracy, and stability of the OSI system in the technical performance tests: an ET Verification Head Phantom (The Phantom Laboratory, Salem, NY, USA) for the head; RS‐1500 (Radiology Support Devices, Inc., Long Beach, CA, USA) for the thorax; and an ET Verification Phantom (The Phantom Laboratory, Salem, NY, USA) for the pelvis. The surfaces of the phantoms were covered with opaque tape in color (RGB:187, 155, 136), which visually resembled human skin tone. For the head phantom, open‐type white and black masks (Engineering System Co., Matsumoto, Nagano, Japan) were prepared.

**Fig. 2:**
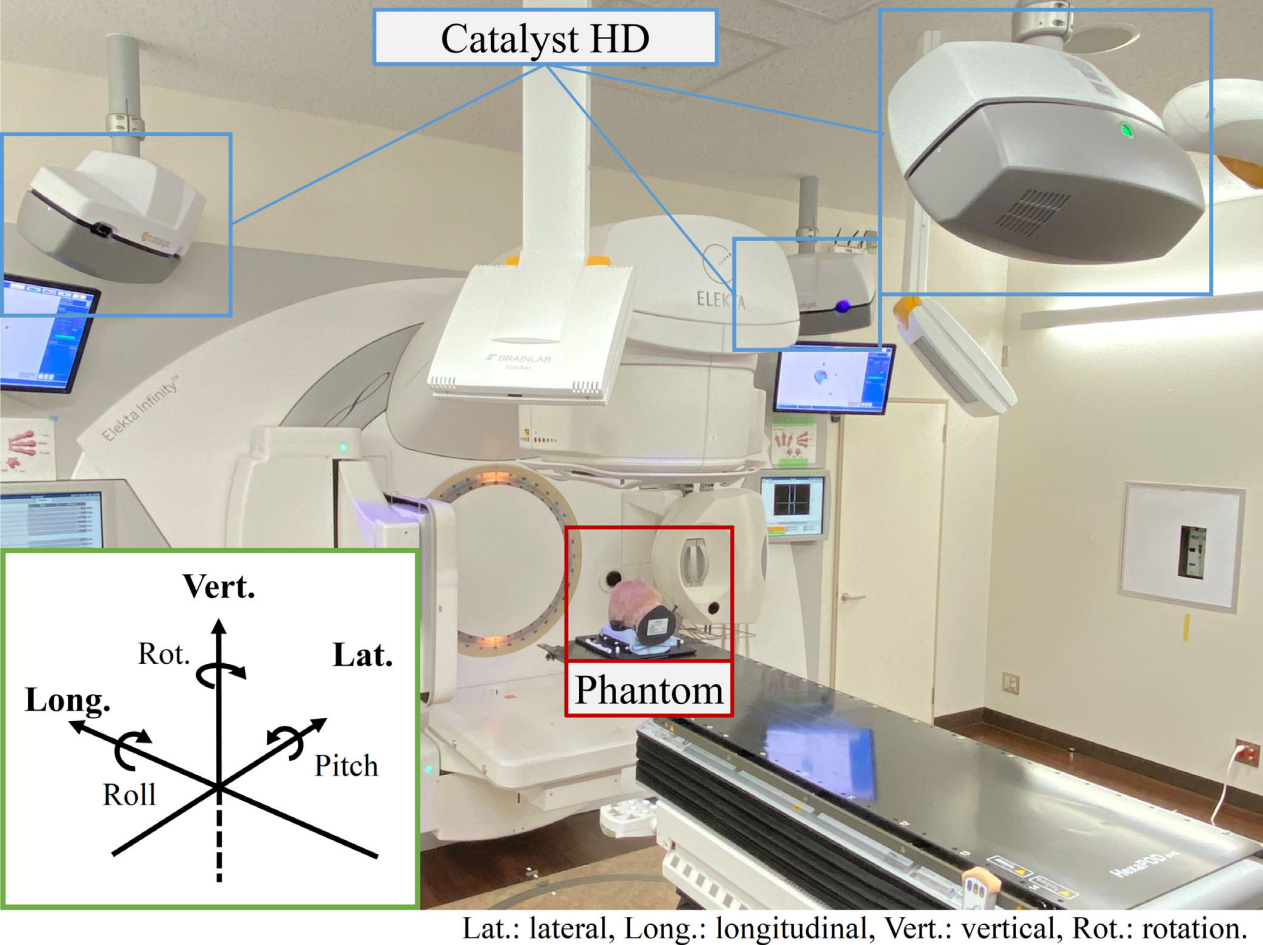
The geometry and coordinate system for the technical performance test. The coordinate system for the technical performance tests was the same as the Elekta Synergy coordinate system: the arrows indicate the positive directions for translation and rotation.

All the technical performance tests were performed using Elekta Infinity (Elekta AB, Stockholm, Sweden), equipped with a six DOF HexaPOD system and an XVI system for cone‐beam computed tomography (CBCT). The gantry angle of the Linac was 0°, and the collimators were completely closed. The ambient room lighting conditions matched typical treatment conditions, although no effect on system accuracy is expected. The orientation was set according to the Elekta Synergy coordinate system and the arrows indicate the positive directions of the coordinate system for translations and rotations (Fig. 2). The phantoms were scanned using a CT system, Aquilion LB (Canon Medical Systems Co., Otawara, Tochigi, Japan), and the CT images of the phantoms were transferred to a treatment planning system, Monaco (Elekta AB, Stockholm, Sweden). A plan was built for each phantom on the Monaco system, and the plan isocenter was located at the center of each phantom. Both the plan and CT images were transferred to the XVI system to rectify the initial position of each phantom.

To determine the reproducibility and accuracy of positioning and stability of motion monitoring of the OSI system, the isocenter displacement was measured. The isocenter displacement was a result of the registration between the reference surface and live surface of the phantoms. The reproducibility, accuracy, and stability values were compared with the phantom types. In addition, they were compared with the algorithms and the mask types, including no mask for the head phantom.

#### 2.B.1 Reproducibility of positioning

**Fig. 3:**
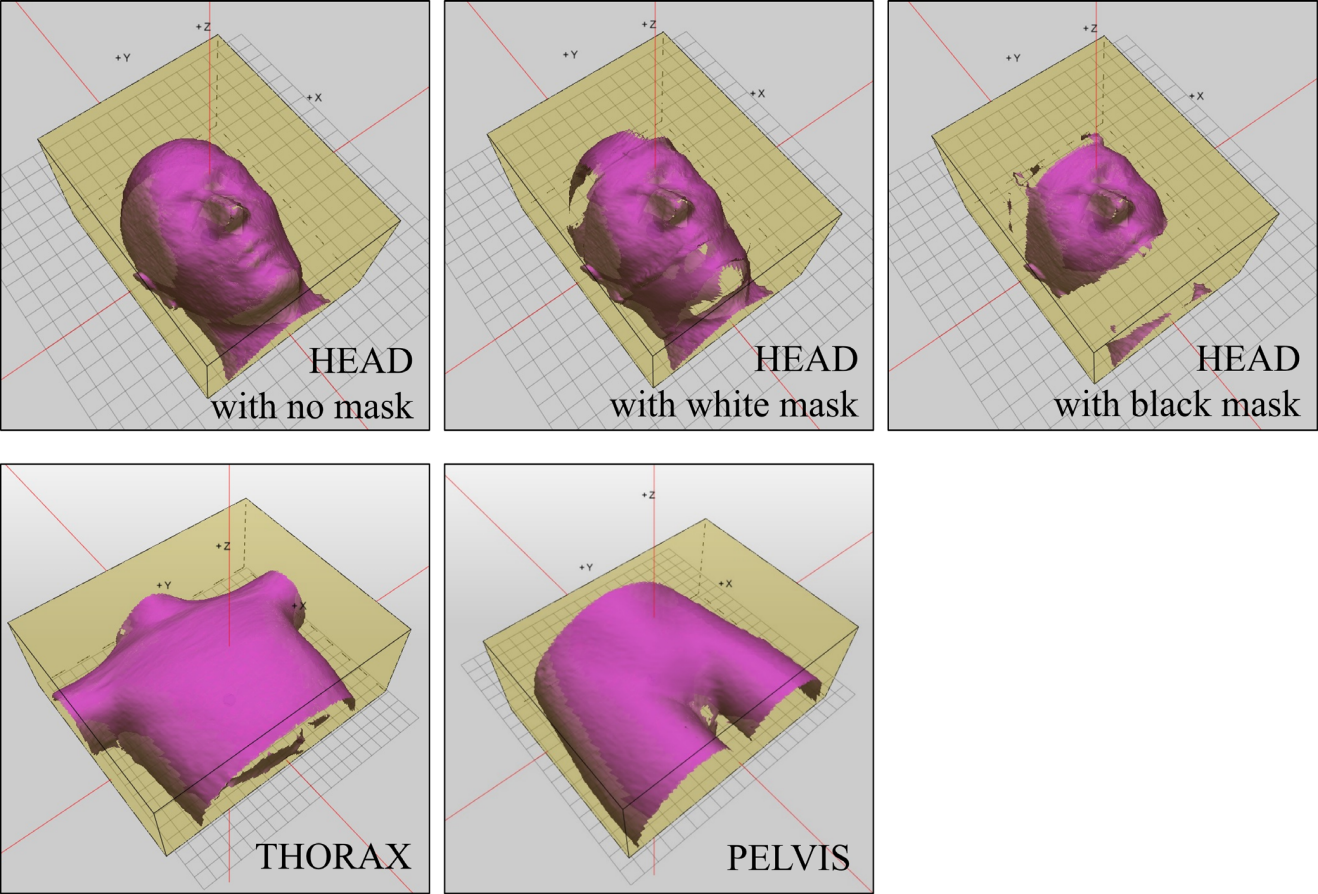
Scan volume and reference surface data for the technical performance test. The yellow boxes depict the scan volume and the reference surfaces are represented in pink.

Reproducibility tests were performed in the positioning mode (cPosition application) of the Catalyst HD system. Each of the head, thorax, and pelvis phantoms were positioned at the isocenter by using CBCT and HexaPOD. Then, each phantom was scanned with the three cameras of Catalyst HD system. The initially scanned surface image was used as a reference surface for reproducibility, and sequentially scanned surface images were registered with the reference image as live images. The scan volume for the live image covered the volume of the entire phantom, and all surface data of the reference surface were also for registration (Fig. 3). The phantoms were not moved during the test, and they were scanned 10 times. The isocenter displacement was calculated immediately after each scan. The means and standard deviations (SDs) of the displacement in six DOFs were calculated. These values were evaluated to determine the reproducibility of the OSI system for interfractional positioning.

The head phantom was immobilized with each of the two masks, and the effect of the masks on the SRS algorithm was compared with that on the conventional algorithm. The settings of the OSI system for the tests are shown in Table 1. The gain and integration time (IT) were set at 300% and 4000 µs for the conventional algorithm and 100% and 18000 µs for the SRS algorithm, respectively.

**Table 1:** The OSI system settings for the technical performance test.

Phantom type	HEAD	THORAX	PELVIS	HEAD (SRS)
Integration time (µs)	4000	4000	4000	18 000
Gain (%)	300	300	300	100
Averaging time (s)	3	4	4	2
Registration HD [high resolution mode]	On	Off	Off	On
Registration algorithm	Conventional (non‐rigid)			SRS (semi‐non‐rigid)

#### 2.B.2 Accuracy of positioning

**Fig. 4:**
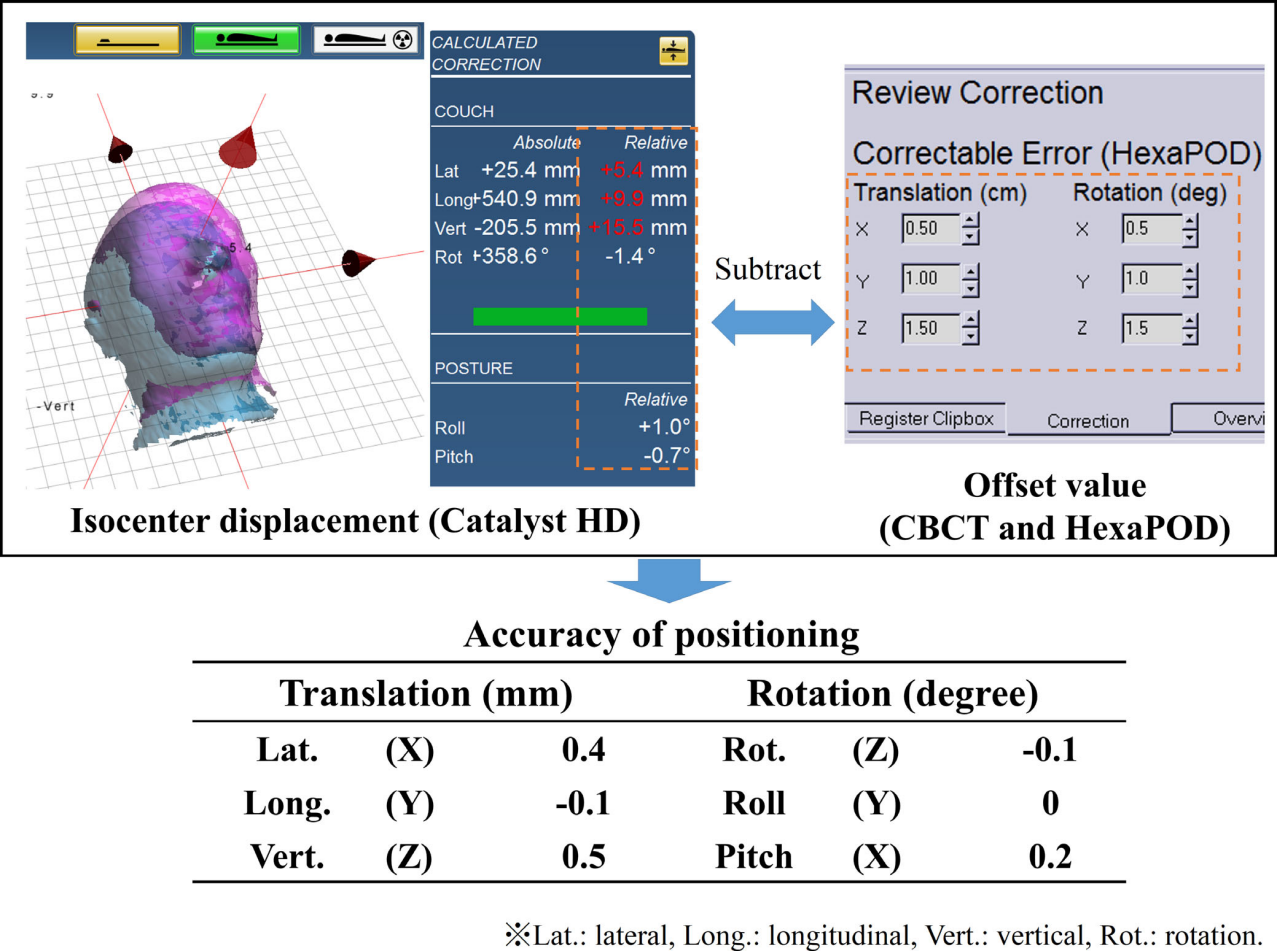
An example of determining the accuracy of positioning. The rotational directions for the Rot. and Pitch of the CBCT and HexaPOD are opposite to those of the Catalyst HD coordinate system.

Accuracy tests were performed in the positioning mode (cPosition application) of the Catalyst HD system. Each of the head, thorax, and pelvis phantoms were positioned at the isocenter, and the position was rectified using CBCT and HexaPOD. Position correction was continued until the residual errors of translation and rotation were close to 0.0 mm and 0.0°, respectively.^8^, ^9^ As in the previous test, each phantom was scanned using the three cameras of the Catalyst HD system and used as a reference surface for the accuracy test. The setup of the scan volume and reference surface data were similar to that used in the reproducibility test (Fig. 3). An offset of 15.0 mm or less for translation and 2.0° or less for rotation were applied to the phantom position by using CBCT and HexaPOD (Table 2). The phantoms were scanned, and the captured phantom surface was registered using the reference surface to calculate the isocenter displacement. These procedures were repeated 10 times, while applying the defined offset to the phantoms for each test. The offset was applied to the head phantom inside the masks. The residual error of correction by Catalyst HD system was calculated by subtracting the offset value from the isocenter displacement (Fig. 4). The means and SDs of the residual error were evaluated to determine the accuracy of the OSI system for interfractional positioning.

**Table 2:** The offset values for translation and rotation for the accuracy test.

	Offset value					
	Translations [mm]			Rotations [°]		
	Lat.	Long.	Vert.	Rot.	Roll	Pitch
Test 1	7.0	7.0	7.0	0.0	0.0	0.0
Test 2	15.0	15.0	15.0	1.0	1.0	1.0
Test 3	−7.0	−7.0	−7.0	0.0	0.0	0.0
Test 4	−15.0	−15.0	−15.0	−1.0	−1.0	−1.0
Test 5	3.0	3.0	3.0	2.0	0.0	0.0
Test 6	−3.0	−3.0	−3.0	−2.0	0.0	0.0
Test 7	3.0	3.0	3.0	0.0	2.0	0.0
Test 8	−3.0	−3.0	−3.0	0.0	−2.0	0.0
Test 9	3.0	3.0	3.0	0.0	0.0	2.0
Test 10	−3.0	−3.0	−3.0	0.0	0.0	−2.0

Lat.: lateral, Long.: longitudinal, Vert.: vertical, Rot.: rotation.

The effect of the masks and the difference in accuracy between the two algorithms were investigated for the head phantom. The settings of the OSI system used in this test are shown in Table 1. The gain and IT were identical to those in the reproducibility test.

#### 2.B.3 Stability of motion monitoring

Stability tests were performed in the monitoring mode (cMotion application) of the Catalyst HD system. Each phantom was positioned in the same way as in the reproducibility test. Then, the application of the Catalyst HD system was switched from the positioning to the monitoring mode. The phantoms were re‐scanned using Catalyst HD system and used as a reference surface for stability. The setups of the scan volume and reference surface data were also identical to those in the other tests (Fig. 3). Each phantom was continuously monitored for 20 min to determine the deviation of isocenter displacement. The monitoring was repeated 10 times, and the means and SDs of the deviation of isocenter displacement in the six DOFs were calculated. These values, which represent the drift of the isocenter location, were evaluated to determine the stability of the OSI system for intrafractional motion monitoring. The head phantom, masks, and settings, including gain and IT, were the same as those in the other tests.

### 2.C Statistical analysis

Multivariate analysis of variance was used for the statistical analysis. The objective variable was the absolute value of isocenter displacement or residual error and the independent variables were the types of the phantoms, algorithms, and mask types, including no mask. The causal relationship between these variable was tested using Wilks’ lambda test with JMP version 13.2.0 (SAS Institute Inc., Cary, NC, USA). The *P* < 0.01 was considered to be statistically significant.

## 3 RESULTS

### 3.A Reproducibility of positioning

The results of the reproducibility test for interfractional positioning are shown in Table 3. All the mean values for translation were <0.1 mm, and those for rotation were <0.1°. There was no significant difference in the reproducibility between the phantom types for the conventional algorithm (*P* = 0.2845). However, the reproducibility of the head phantom for the SRS algorithm was significantly higher than that of the other phantoms for the conventional algorithm (*P* < 0.01).

**Table 3:** Results of the reproducibility test for interfractional positioning.

Phantom	Mask	Translations: mean (SD) [mm]			Rotations: mean (SD) [°]		
		Lat.	Long.	Vert.	Rot.	Roll	Pitch
Head	No	0.01 (0.06)	0.04 (0.05)	−0.09 (0.03)	−0.01 (0.03)	0.03 (0.05)	0.01 (0.03)
	White	0.04 (0.05)	−0.02 (0.04)	−0.07 (0.07)	−0.01 (0.03)	−0.02 (0.04)	0.01 (0.03)
	Black	0.07 (0.08)	−0.08 (0.08)	0.04 (0.05)	0.01 (0.03)	−0.05 (0.05)	−0.05 (0.07)
Thorax		0.06 (0.07)	−0.04 (0.05)	−0.07 (0.05)	0.01 (0.03)	−0.02 (0.04)	0.01 (0.03)
Pelvis		0.09 (0.05)	−0.09 (0.07)	−0.08 (0.09)	0.02 (0.04)	−0.05 (0.05)	−0.02 (0.04)
Head (SRS)	No	0.01 (0.03)	−0.02 (0.04)	0.02 (0.04)	−0.01 (0.03)	0.01 (0.03)	0.01 (0.03)
	White	−0.02 (0.04)	−0.02 (0.04)	0.01 (0.03)	−0.01 (0.03)	0.02 (0.04)	−0.01 (0.03)
	Black	0.02 (0.04)	−0.01 (0.03)	−0.01 (0.03)	0.01 (0.03)	0.01 (0.03)	−0.01 (0.03)

SD: standard deviation, Lat.: lateral, Long.: longitudinal, Vert.: vertical, Rot.: rotation.

**Fig. 5:**
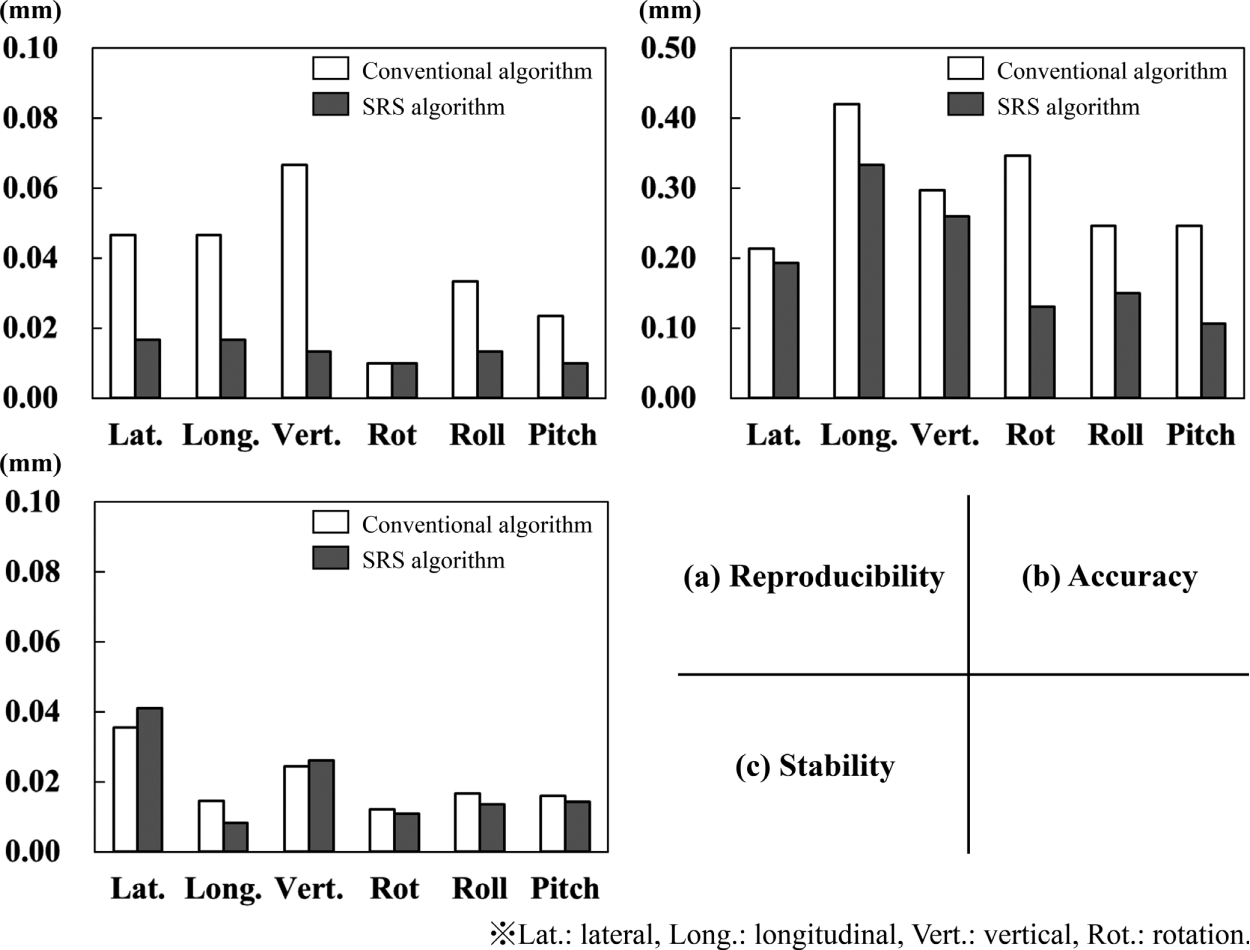
The results of reproducibility, accuracy, and stability for the head phantom using the conventional and SRS algorithms. These results show the mean absolute error of isocenter displacement or residual error.

Regarding the head phantom, the results of reproducibility showed no significant difference among the tests with no mask, those with white masks, and those with black masks for the conventional algorithm (*P* = 0.5689) and the SRS algorithm (*P* = 0.9758). A significant difference was observed between the two algorithms (*P* < 0.01). The maximum SDs for translation and rotation for the conventional algorithm were 0.08 mm and 0.07°, respectively, while those for the SRS algorithm were 0.04 mm and 0.04°, respectively. The mean absolute error of reproducibility for the conventional and SRS algorithms is shown in Fig. 5(a). The SRS algorithm had a small SD and mean absolute error. Thus, the SRS algorithm has a higher reproducibility than the conventional algorithm.

### 3.B Accuracy of positioning

The results of the accuracy test for interfractional positioning are shown in Table 4. All the mean values for translation were <1.0 mm, and those for rotation were <1.0°. For the conventional algorithm, the accuracy of the pelvis phantom was significantly lower than that of the other phantom types (*P* < 0.01), and the mean value for the translation of the pelvis in the longitudinal direction was 0.90 ± 0.80 mm. The accuracy of the head phantom for the SRS algorithm was significantly higher than that of the other phantoms for the conventional algorithm (*P* < 0.01).

**Table 4:** Results of the accuracy test for interfractional positioning.

Phantom	Mask	Translations: mean (SD) [mm]			Rotations: mean (SD) [°]		
		Lat.	Long.	Vert.	Rot.	Roll	Pitch
Head	No	0.06 (0.10)	0.36 (0.27)	−0.27 (0.17)	−0.02 (0.39)	−0.29 (0.24)	0.17 (0.24)
	White	−0.42 (0.28)	0.48 (0.30)	0.39 (0.25)	0.34 (0.33)	−0.02 (0.31)	−0.17 (0.33)
	Black	0.04 (0.17)	0.02 (0.49)	−0.21 (0.16)	0.00 (0.50)	−0.09 (0.23)	0.08 (0.31)
Thorax		0.00 (0.27)	0.25 (0.18)	−0.09 (0.37)	0.00 (0.16)	−0.03 (0.31)	0.04 (0.27)
Pelvis		−0.19 (0.14)	0.90 (0.80)	−0.24 (0.32)	0.02 (0.19)	−0.14 (0.34)	−0.11 (0.17)
Head (SRS)	No	−0.13 (0.07)	0.38 (0.18)	0.00 (0.07)	0.00 (0.11)	−0.15 (0.14)	0.08 (0.08)
	White	−0.08 (0.18)	0.24 (0.30)	0.51 (0.38)	0.00 (0.25)	−0.16 (0.14)	0.14 (0.23)
	Black	−0.31 (0.11)	0.20 (0.27)	0.23 (0.13)	0.13 (0.08)	−0.12 (0.10)	0.08 (0.06)

SD: standard deviation, Lat.: lateral, Long.: longitudinal, Vert.: vertical, Rot.: rotation.

The results for the head phantom showed no significant difference for tests conducted with no mask, the white mask, and the black mask for the conventional algorithm (*P* = 0.0381); however, a significant difference was observed for the SRS algorithm (*P* < 0.01). In particular, for the SRS algorithm, the variation in the isocenter displacement for the white mask was greater than those for without a mask and the black mask for all values of translation and rotation. There was also a significant difference between the two algorithms (*P* < 0.01). The mean absolute error of accuracy for the conventional and SRS algorithms is shown in Fig. 5(b). The SRS algorithm had a small mean absolute error. Thus the SRS algorithm had a higher accuracy than the conventional algorithm.

### 3.C Stability of motion monitoring

The results of the stability of intrafractional monitoring are shown in Table 5. All the mean values for translation were <0.1 mm, and those for rotation were <0.1°. There was a significant difference in the stability between the phantom types (*P* < 0.01).

**Table 5:** Results of the stability test for intrafractional motion monitoring.

Phantom	Mask	Translations: mean (SD) [mm]			Rotations: mean (SD) [°]		
		Lat.	Long.	Vert.	Rot.	Roll	Pitch
Head	No	−0.04 (0.02)	0.01 (0.02)	−0.01 (0.02)	0.01 (0.01)	0.01 (0.01)	−0.01 (0.01)
	White	0.02 (0.03)	−0.01 (0.01)	0.00 (0.02)	0.01 (0.01)	−0.01 (0.01)	0.01 (0.02)
	Black	0.03 (0.03)	−0.00 (0.02)	0.02 (0.02)	−0.01 (0.01)	−0.01 (0.02)	0.02 (0.01)
Thorax		−0.01 (0.01)	0.03 (0.01)	−0.03 (0.02)	0.00 (0.01)	0.01 (0.01)	0.02 (0.01)
Pelvis		0.00 (0.02)	0.05 (0.02)	−0.02 (0.01)	−0.01 (0.01)	0.00 (0.01)	0.02 (0.01)
Head (SRS)	No	−0.04 (0.01)	0.00 (0.01)	−0.03 (0.01)	0.01 (0.01)	0.01 (0.01)	0.01 (0.01)
	White	0.05 (0.01)	−0.00 (0.01)	−0.03 (0.01)	−0.01 (0.01)	0.01 (0.01)	0.01 (0.03)
	Black	−0.04 (0.01)	−0.01 (0.01)	−0.02 (0.01)	0.01 (0.01)	0.01 (0.01)	−0.01 (0.01)

SD: standard deviation, Lat.: lateral, Long.: longitudinal, Vert.: vertical, Rot.: rotation.

The stability for the head phantom showed no significant difference among the tests conducted with no mask, the white mask, and the black mask for the conventional (*P* = 0.6940) and SRS (*P* = 0.1639) algorithms. The mean absolute error of stability for the conventional and SRS algorithms is shown in Fig. 5(c). The stability was found not to be significantly affected by the algorithms (*P* = 0.0280).

## 4 DISCUSSION

The reproducibility, accuracy, and stability of the Catalyst HD system were investigated using rigid phantoms. The reproducibility for translation was <0.1 mm and that for rotation was <0.1°, for all sites and both algorithms. A similar study, using the Sentinel system,^2^ reported that the reproducibility for translation was <0.5 mm and that for the rotation was <0.1°. The Sentinel system acquires surface images by using multiple line scans with a laser, while the Catalyst HD system has three scanner units and acquires surface images with a wide coverage by using one entire projection with a light emitting diode. In this study, the differences in surface scanning methods and surface image coverage led to higher reproducibility for the Catalyst HD system than for the Sentinel system. In addition, the nominal reproducibility of Catalyst HD system, which C‐RAD published on the company website, was 0.2 mm,^10^ which is comparable to our results.

The accuracy for translation was <1.0 mm and that for rotation was <1.0°, for all sites and both algorithms. Several previous studies using phantoms^2^, ^3^, ^11^ reported that the accuracy was <1.0 mm and the nominal accuracy of Catalyst HD system, which C‐RAD published on the company website, was ≤0.5 mm for a rigid body,^10^ which is comparable to our results. The accuracy for the pelvis phantom was significantly worse in the longitudinal direction. A previous clinical study^5^ reported that the accuracy for the pelvis was 0.1, 1.8, and 1.4 mm in the lateral, longitudinal, and vertical directions, respectively, which is comparable to our results.

**Fig. 6:**
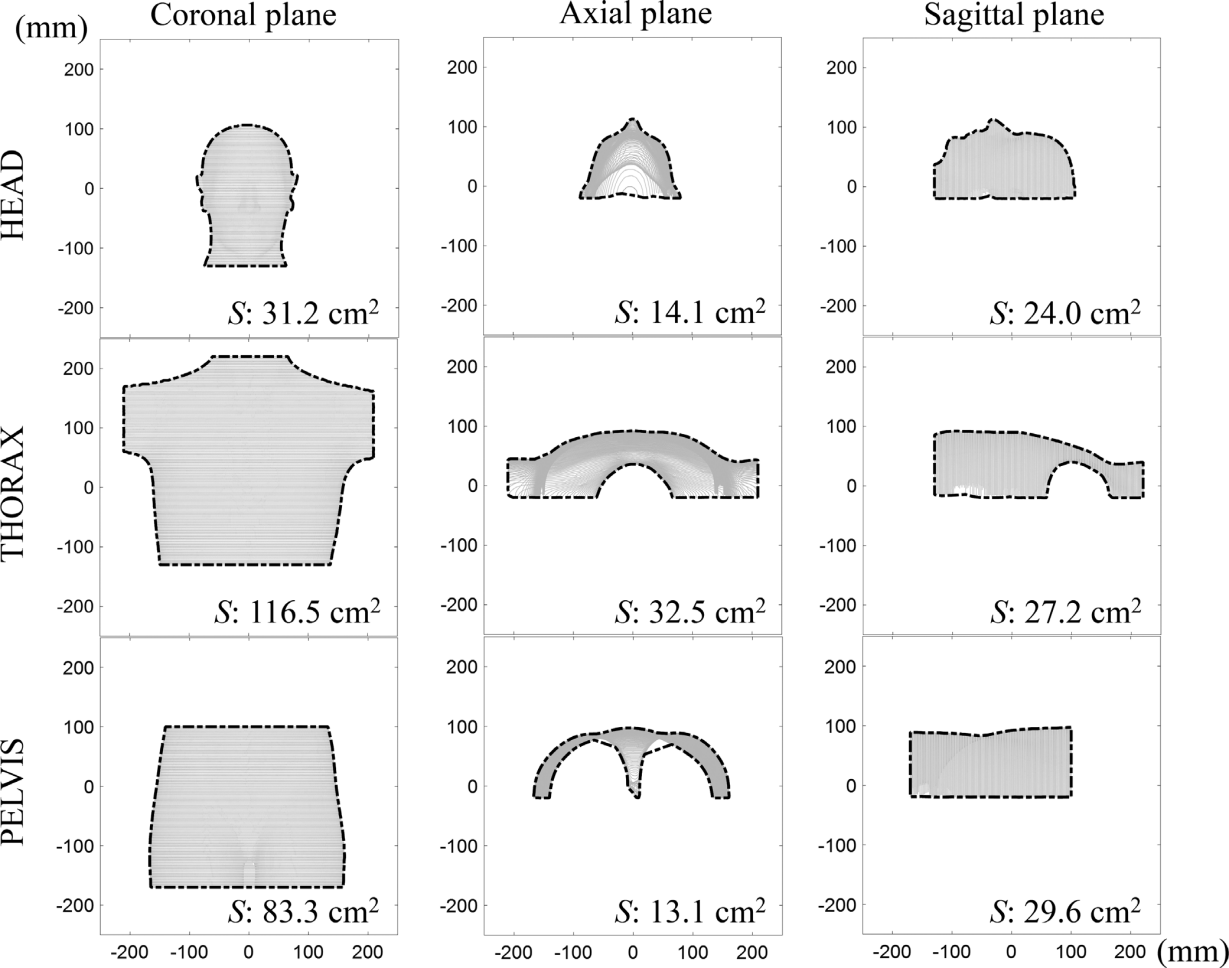
Projected reference surface of the phantoms onto coronal, axial, and sagittal planes. *S* denotes the area value of the projected phantom in each plane image.

We believe that the additional inaccuracy along the longitudinal direction was primarily because the surface of the pelvis phantom was more cylindrically symmetric than that of the other phantoms. In other words, the isometry of the edges of the source surface images in the non‐rigid ICP registration was not maintained correctly along the longitudinal direction.^7^ Therefore, the penalties of registration calculation owing to the translations along the longitudinal axis were not as accurate as those along the other axes. As an alternative approach, we believe that the size of the surface area perpendicular to the longitudinal axis may affect the accuracy of registration calculation. Therefore, three‐dimensional points, comprising the reference surface for accuracy, were plotted on a two‐dimensional coronal, axial, and sagittal plane, perpendicular to the axis of the vertical, longitudinal, and lateral direction, respectively. A boundary around the points was computed. The area inside the boundary of each plane was calculated for the head, thorax, and pelvis phantoms (Fig. 6). The pelvis phantom had a smaller area value on the axial plane than the other phantoms. The absolute area value on the axial plane was 14.1 and 32.5 cm^2^ for the head and thorax phantoms, respectively, while it was only 13.1 cm^2^ for the pelvis phantom. Owing to the very small surface area, the warping of the source surface to the target surface during the registration process could have been inaccurate, and a penalty for the calculation of the non‐rigid ICP registration could have been incorrectly assigned.^7^ Based on the above discussion, the low positioning accuracy for the pelvis phantom in the longitudinal direction could be attributed to the shape and size of the reference surface image. The relationship between the positioning accuracy and the shape and size of the reference surface requires further investigation.

The stability for translation was <0.1 mm and that for rotation was <0.1° for all sites and both algorithms. The nominal stability of Catalyst HD system, which C‐RAD published on the company website, was ≤0.5 mm^10^; this is comparable to our results. Although the Catalyst HD system may have sufficient stability to detect patient motions in high‐precision radiation therapy, such as intensity modulated radiation therapy and SRS, its stability may deteriorate when a gantry and an imaging device obstruct its scanner unit and when a Linac couch rotates.^12^ Further, the ability to detect the isocenter displacement in motion monitoring (i.e., the accuracy of motion monitoring) was inferred to be comparable to the positioning accuracy results obtained under the same settings for the Catalyst HD system. With respect to the monitoring mode, this was because the surface scanned by Catalyst HD system was always used as the reference surface for the registration.

The SRS algorithm demonstrated better reproducibility and accuracy than the conventional algorithm for the head phantom. This was because the reconstructed surface image obtained using the SRS algorithm had a higher image resolution. Moreover, the uncertainty of the registration calculation was reduced because of an increase in the number of corresponding point pairs between the live surface and the reference surface. In contrast, the increase in the number of their points for the registration may take longer computation time. Therefore, it is better for the SRS algorithm to choose less deformable and smaller areas of anatomical sites for registration.

Another aspect is that the white mask might be recognized as a part of the head, which may lead to errors in calculating the isocenter displacement, owing to the misalignment between the mask itself and the inside of the mask. In particular, the SRS algorithm using semi‐non‐rigid registration, which is not good for dealing with deformation, may result in larger errors than the conventional algorithm using non‐rigid registration. Therefore, for treatment to the head and face using the SRS algorithm, the black mask that is undetectable by Catalyst HD system may be more suitable than the white mask. In addition, a previous study^13^ reported that the difference in the positioning accuracy between the SRS with an open‐type mask using another OSI system, and the SRS with a closed‐type mask using the infrared tracking system was ≤1.0 mm for translation and ≤1.0° for rotation. The accuracy of this study is comparable to, or better than, that of the previous clinical study^13^ for SRS with an open‐type white mask using another hardware (AlignRT system). We suggest that an open‐type black mask should be worn when using the SRS algorithm for the head and face during clinical use.

A limitation of this study is that the results presented in this paper were obtained using rigid body phantoms. The results may not fully reflect the technical performance of this system in a clinical situation. Several uncertainties, including patient’s body shape changes, respiratory movement, and the reference surface size (scan volume) and type (external contour of a treatment plan or scan by the OSI system) will probably affect the technical performance of the system in clinical use. In particular, some uncertainties related to the contouring process (e.g., differences in size of voxel size, number of contour composition points, and operators) may further affect the technical performance when using the external contour of the treatment plan as a reference surface. The sensitivity and technical performance tests in this study were performed using the Catalyst HD system only. OSI systems released from other companies may not achieve the same results.

## 5 CONCLUSION

In this study, we evaluated the reproducibility and accuracy of interfractional patient positioning and the stability of intrafractional motion monitoring. The reproducibility for translation and rotation was <0.1 mm and <0.1°, respectively; the accuracy was <1.0 mm and <1.0°, respectively; and the stability was <0.1 mm and <0.1°, respectively. In particular, the SRS algorithm had a significantly higher reproducibility and accuracy than the conventional algorithm (*P* < 0.01), and a small absolute error and SD of calculated isocenter displacement. There was no difference in the stability between the algorithms (*P* = 0.0280). The SRS algorithm was found to be suitable for the treatment of rigid body sites with less deformation and small area, such as the head and face.

## AUTHORS CONTRIBUTIONS

Hironori Kojima: Conceptualization, Formal analysis, Investigation, Software, Writing – original draft (lead), and Writing – review and editing. Akihiro Takemura: Methodology, Project administration, Validation, Writing – original draft (support), and Writing – review and editing. Shogo Kurokawa: Data curation, Formal analysis, Investigation, Visualization, and Writing – review and editing. Shinichi Ueda: Data curation, Investigation, Project administration, Visualization, and Writing – review and editing. Kimiya Noto: Data curation, Validation, Writing – review and editing. Haruna Yokoyama: Data curation, Investigation, Visualization, and Writing – review and editing. Shigeyuki Takamatsu: Methodology, Project administration, Supervision, and Writing – review and editing.

## CONFLICT OF INTEREST

No conflict of interest.
